# Comparison of the Effectiveness of the C‐MAC Versus the McGrath Videolaryngoscope for Adult Endotracheal Intubation in Clinical Patients, Simulated Patients, and Manikin Settings: A Systematic Review and Meta‐analysis

**DOI:** 10.1155/anrp/1575867

**Published:** 2026-08-31

**Authors:** Evgenia Dimou, Diamanto Aretha, Theano Beskou-Kontou, Antonis Kostouros, Matthaios Pappas, Angeliki Panagopoulou, Theodoros Xanthos

**Affiliations:** ^1^ Department of Anesthesiology and Intensive Care, University Hospital of Patras, Patras 26504, Greece, pgnp.gr; ^2^ School of Health Sciences, University of West Attica, Athens 10434, Greece, uniwa.gr

**Keywords:** C-MAC, McGrath, meta-analysis, systematic review, videolaryngoscopes

## Abstract

**Introduction:**

Videolaryngoscopes are widely utilized in daily clinical practice within anesthesiology and emergency medicine to enhance airway management techniques and promote patient safety. Among these devices, the C‐MAC and McGrath videolaryngoscopes have been extensively evaluated in multiple studies to determine their relative efficacy. This systematic review aims to synthesize the findings of available research comparing the C‐MAC and McGrath with respect to first‐pass intubation success and intubation time, as well as to conduct a meta‐analysis for statistical evaluation.

**Materials and Methods:**

A systematic review was performed using PubMed, Scopus, Cochrane Library, and Google Scholar in accordance with PRISMA guidelines. Eligible studies directly compared the C‐MAC and McGrath videolaryngoscopes in adult clinical patients, simulated‐patient clinical scenarios, or adult manikin–based airway models. Because these populations are methodologically and clinically heterogeneous, adult clinical patient studies were strictly separated from simulated‐patient and manikin studies. Quantitative meta‐analysis was restricted to adult clinical patient studies, whereas simulated‐patient and manikin studies were summarized qualitatively. Studies were screened up to June 23, 2025, and assessed for bias with the Cochrane Risk of Bias 2.0 tool. Results are summarized in tables, and meta‐analysis was conducted using R (meta‐package, V4.4.1), focusing on first‐pass intubation success and intubation time, displayed as forest plots. Certainty of evidence for the main outcomes was assessed using a GRADE‐based approach, while publication bias and sensitivity analyses were also performed.

**Results:**

Nine studies met the inclusion criteria. The evidence base comprised six adult clinical patient studies and three manikin‐based airway studies. Adult clinical patient studies and manikin‐based studies were reported separately, and manikin datasets were not counted as clinical patients. Six adult clinical patient studies were included in the quantitative meta‐analysis, whereas manikin‐based studies were summarized qualitatively. No definitive superiority of either device was demonstrated for first‐pass intubation success in adult clinical patients. C‐MAC showed shorter intubation time in some analyses, but heterogeneity was substantial, and certainty of evidence was limited.

**Discussion:**

The available evidence should be interpreted cautiously because of clinical heterogeneity, differences in airway difficulty, operator experience, device blade configuration, and outcome definitions. Simulated‐patient and manikin‐based airway studies provide useful information on device handling and training but should not be interpreted as directly equivalent to adult clinical patient evidence.

## 1. Introduction

Effective airway management is central to anesthesiology and emergency medicine, where endotracheal intubation is commonly used to secure the airway [1].

Although direct laryngoscopy is usually effective, failed intubation can cause serious complications. Videolaryngoscopes were developed to improve glottic visualization and intubation conditions. Videolaryngoscopy improves first‐pass success and reduces complications such as hypoxemia, airway trauma, and unrecognized esophageal intubation [2]. In failed standard intubation, videolaryngoscopy has shown high rescue success, including a 92% success rate in a multicenter retrospective study [3], while a Cochrane review by Lewis et al. found fewer failed intubations with videolaryngoscopy than with direct laryngoscopy, particularly in anticipated difficult airways [4].

A difficult airway is generally defined as difficulty with mask ventilation or tracheal intubation by a trained anesthesiologist [5], and DAS and ASA guidance emphasizes that device selection should reflect the clinician’s training and experience [6].

Videolaryngoscopes also facilitate intubation in morbid obesity [7], although routine use in this group remains inconsistent despite supportive evidence and guidelines [8]. A recent meta‐analysis reported improved first‐pass success with videolaryngoscopy versus direct laryngoscopy in obese patients without prolonging intubation time [9].

SARS‐CoV‐2 spreads easily and can cause severe lung complications as well as serious systemic issues affecting organs throughout the body [10]. Airway management for COVID‐19 patients prioritizes safety and recommends videolaryngoscopy as the primary method for tracheal intubation, included in all airway kits according to recent clinical guidelines [11]. DAS‐led consensus guidance recommended videolaryngoscopes, particularly those with remote screens, to improve first‐attempt success and reduce exposure risk when used by experienced operators [12]. Evidence in COVID‐19 airway scenarios remains mixed: Biro et al. found shorter glottic visualization but longer airway management with nonchanneled devices [13], whereas Jayaram et al. reported better success and shorter times with Airtraq than McGrath [14]. Other studies found no clear superiority between channeled and nonchanneled videolaryngoscopes [15], including postpandemic data, highlighting the need for further research [16].

In trauma, factors such as bleeding, maxillofacial injury, cervical spine immobilization, and hypoxemia increase intubation risk [17, 18]. The use of videolaryngoscopy is thus increasingly favored for securing the airway of polytrauma patients [19], supporting wider use of videolaryngoscopy despite limitations such as lens contamination and cost [20].

Among available devices, the C‐MAC and McGrath videolaryngoscopes are widely studied [21, 22]. However, it remains unclear which performs better for adult endotracheal intubation under general anesthesia. This review synthesizes published evidence comparing their clinical performance.

## 2. Methods

### 2.1. Design and Inclusion–Exclusion Criteria

This systematic review was registered prospectively in PROSPERO (CRD420251250283) https://www.crd.york.ac.uk/PROSPERO/view/CRD420251250283. The review was conducted in accordance with the Preferred Reporting Items for Systematic Reviews and Meta‐Analyses (PRISMA) guidelines [23] (Supporting Information), based on predefined inclusion criteria established by the authors. Two reviewers independently screened abstracts after conducting an English‐language literature search. Inclusion was based on prespecified criteria. A full PRISMA checklist is provided in the Supporting Information to ensure transparency, along with a list of abbreviations explained (Nomenclature).

The review question was structured according to the PICO framework. The population comprised adult clinical patients undergoing endotracheal intubation, simulated‐patient clinical scenarios, and manikin‐based adult airway models. The intervention was intubation with either the C‐MAC or McGrath videolaryngoscope, and the comparator was the alternative device. The primary outcomes were first‐pass intubation success and time to intubation; secondary outcomes included total intubation success, number of attempts, glottic view, optimization maneuvers, operator difficulty or preference, and reported airway complications.

Studies were eligible for inclusion in the systematic review only if they met all of the following criteria: (1) randomized controlled trial, prospective randomized study, or randomized crossover design; (2) adult clinical patients undergoing endotracheal intubation, simulated‐patient clinical scenarios, or manikin‐based adult airway models; (3) direct comparison between the C‐MAC and McGrath videolaryngoscopes within the same study; (4) reporting of at least one prespecified outcome, including first‐pass intubation success, time to intubation, overall intubation success, number of attempts, glottic view, optimization maneuvers, operator difficulty or preference, or airway complications; and (5) availability of sufficient methodological and outcome data for qualitative synthesis. For the quantitative meta‐analysis, eligibility was further restricted to adult clinical patient studies with extractable numerical data for first‐pass intubation success and/or time to intubation.

Studies were excluded if they met any of the following criteria: (1) pediatric population; (2) animal study; (3) nonrandomized observational design; (4) case report, case series, editorial, narrative review, or conference abstract without full data; (5) comparison that did not include both C‐MAC and McGrath; (6) comparison of only one of the two devices with the conventional Macintosh laryngoscope; (7) insufficient outcome data for the prespecified endpoints; or (8) exclusive use of simulated‐patient clinical scenarios or manikin‐based airway models when quantitative pooling was considered. Simulated‐patient and manikin‐based studies were retained for qualitative synthesis when they otherwise met the systematic review criteria, but they were not included in the quantitative meta‐analysis because they are not clinically equivalent to adult clinical patient studies.

### 2.2. Database Search and Study Selection

The databases PubMed, Google Scholar, Scopus, and the Cochrane Library were used for the primary literature search, supplemented by screening the reference lists of electronically retrieved articles (snowballing). The last search was conducted on June 23rd, 2025. The search strategy employed a combination of keywords and Boolean operators to ensure precise retrieval of relevant studies. The following terms were used in our search strategy:

((“Videolaryngoscopy”[MeSH] OR “Videolaryngoscopy/standards”[MeSH] OR “Laryngoscopes”[MeSH]) AND “Tracheal Intubation”[MeSH] AND “Airway Management”[MeSH]) AND (“C‐MAC”[tiab] OR “C‐MAC videolaryngoscope”[tiab] OR “C‐MAC D‐Blade”[tiab]) AND (“McGrath”[tiab] OR “McGrath MAC”[tiab]) NOT (“Animal Study”[MeSH] OR “Case Report”[Publication Type] OR “Pediatric Patients”[MeSH]).

After searches were completed, retrieved articles were exported into EndNote (X9.3.3 for Windows) for the removal of duplicates. Study selection according to inclusion–exclusion criteria was independently conducted by two authors (D.E and A.D) according to the PRISMA guidelines [23]. Discrepancies between reviewers were discussed until consensus was reached. The study selection process is depicted in the PRISMA flow diagram.

### 2.3. Data Extraction and Assessment of Methodological Quality and Risk of Bias

Two authors (D.E and A.D) independently extracted data from the included studies by using a standardized data collection form, which included the following:

a. Study characteristics: the study’s unique identifier (DOI) listed in ascending numerical order, first author’s name, publication year, journal name, study design, total participant count, main intervention studied, and any noted funding sources or conflicts of interest. Additionally, details reported may cover degree of airway difficulty, operator experience level, type of laryngoscopes used, as well as each study’s defined primary and secondary outcomes.
b. Study findings: in ascending numerical order and according to the DOI of each study, the results for the primary and secondary outcomes of all included studies.

Risk of bias of the included studies was appraised by the Cochrane Risk of Bias 2.0 tool for randomized controlled trials [24]. The Risk of Bias tool assesses potential bias in five domains: randomization, intervention assignment and adherence, missing outcome data, outcome measurement, and selection of reported results. Each domain is rated as “low risk,” “some concerns,” or “high risk” using structured signaling questions. Overall risk judgments are based on these domain ratings for a transparent review. If reviewers disagreed, articles were re‐examined until consensus was reached.

Certainty of evidence for the main outcomes was assessed using a GRADE‐based approach. Evidence from randomized clinical studies was initially considered high certainty and was downgraded where appropriate for risk of bias, inconsistency, indirectness, imprecision, or suspected publication bias. Certainty judgments were summarized in a dedicated summary‐of‐findings table.

### 2.4. Data Synthesis and Analysis

Study characteristics, findings, risk of bias, and certainty of evidence were summarized in tables and within the text. Quantitative synthesis was restricted to adult clinical patient studies to avoid inappropriate pooling of adult clinical patient, simulated patient, and manikin‐based data. The meta package in R Version 4.4.1 was used for analysis. The metabin function was applied for binary data, and metacont was used for continuous data. Results were illustrated as forest plots. Odds ratios (ORs) and 95% confidence intervals were calculated for first‐pass intubation success, and mean differences with 95% confidence intervals were calculated for intubation time. Heterogeneity was assessed using I^2^, tau^2^, H statistic, and the *Q* test. Fixed‐effect models were used when heterogeneity was low, whereas random‐effects models were prioritized when heterogeneity was moderate or high. Funnel plots and Egger’s tests were interpreted cautiously because fewer than 10 studies were available. Sensitivity analyses were conducted by excluding studies with differing primary outcomes.

## 3. Results

### 3.1. Study Selection Process

**FIGURE 1:**
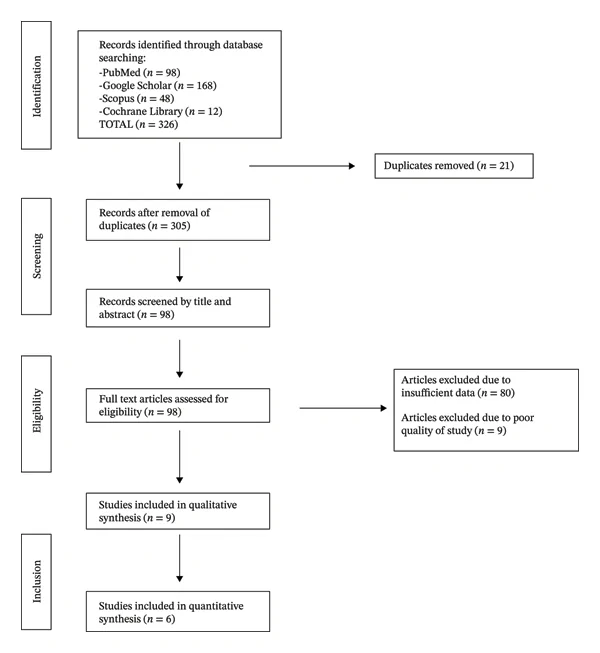
The PRISMA flowchart.

A total of 326 records were identified across the databases using the search algorithm and eligibility criteria described above. After removing 21 duplicates, 305 studies remained for title and abstract screening. Of these, 207 were excluded as irrelevant to the topic or not meeting the inclusion criteria (e.g., studies involving aediatric populations, nonrandomized designs, or studies comparing either of the two videolaryngoscopes with the conventional Macintosh laryngoscope). The remaining 98 articles were assessed in full text. Of these, 80 were excluded due to insufficient data and 9 due to poor study quality. Ultimately, 9 studies met the inclusion criteria and were included in the review (Figure 1)

### 3.2. Study Design, Data Collection, and Bias Assessment

The characteristics of the included studies [25–33] are presented in Tables 1 and 2.

**TABLE 1:** Demographic characteristics of the studies.

A/n:	DOI	Name of the 1st author	Publication year	Published journal	Study design	Number of participants	Intervention	Funding/conflicts of interest
1.	10.1089/bari.2018.0052	Akbas, Sedat	2019	Bariatric Surgical Practice and Patient Care	RCT	80	Intubation with C‐MAC and McGrath in morbid obesity	No funding; no conflicts of interest
2.	10.5152/TJAR.2021.21251	Gupta, Nishkarsh	2022	Turkish Journal of Anesthesiology and Reanimation	Prospective randomized study	60	COVID‐19 scenario: intubation with C‐MAC and McGrath using an intubation box	No funding; no conflicts of interest
3.	10.1093/bja/aew058	Kleine‐Brueggeney, Maren	2016	British Journal of Anesthesia	Multicenter RCT	720	Intubation using 3 channeled and 3 nonchanneled videolaryngoscopes	Funded by the Gottfried and Julia Bargerter‐Rhyner Foundation et al.; no conflicts of interest
4.	10.1093/bja/aes145	Ng, Irene	2012	British Journal of Anesthesia	RCT	130	Intubation with C‐MAC and McGrath in patients with Mallampati ≥ 3	Funded by local grants; no conflicts of interest
5.	10.1111/anae.13413	Pieters, B.M.A.	2016	Anesthesia	Prospective randomized study	253	Manikin intubation with 7 different devices performed by anesthesiologists, residents, medical students, and paramedical personnel	Funded by departmental grants; no conflicts of interest
6.	10.1155/2016/8943931	Shin, Myungju	2016	BioMed Research International	Randomized crossover manikin study	39	Manikin intubation in normal and difficult airway conditions (tongue edema) performed by inexperienced medical students using C‐MAC, McGrath, and the standard Macintosh laryngoscope	No funding; no conflicts of interest
7.	10.4103/sja.sja_1058_20	Vig, Saurabh	2021	Saudi Journal of Anesthesia	Randomized crossover trial	30	COVID‐19 simulation using an intubation box on a manikin, with C‐MAC and McGrath used by inexperienced physicians after brief videolaryngoscope training	No funding; no conflicts of interest
8.	10.1016/j.jclinane.2015.12.042	Yumul, Roya	2016	Journal of Clinical Anesthesia	RCT	121	Intubation of patients with BMI > 30 undergoing bariatric surgery using a conventional laryngoscope, the Video‐MAC, and the McGrath VL	Funded by the Cedars‐Sinai Medical Center, Department of Anesthesiology, Los Angeles, CA; no conflicts of interest
9.	10.1007/s10877‐021‐00746‐5	Zhang, Jinbin	2021	Journal of Clinical Monitoring and Computing	Two‐center RCT	210	Intubation with MILS maneuver using either the McGrath X‐blade or the C‐MAC VL	Funded by the Alexandra Health Enabling Grant; no conflicts of interest

**TABLE 2:** Clinical characteristics of the studies.

A/n:	DOI:	Airway difficulty	Operator experience	Type of laryngoscope	Primary endpoints:	Secondary endpoints
1.	10.1089/bari.2018.0052	BMI ≥ 40 kg/m^2^	Experienced consultant anesthesiologists	McGrath vs. C‐MAC blade no. 4	Time to intubation and intubation success	Cormack–Lehane (C‐L) grade, POGO score, ease of intubation, hemodynamic response, patient positioning, adverse events
2.	10.5152/TJAR.2021.21251	Exclusion of patients with risk factors for difficult ventilation and airway. Difficulty due to the presence of an intubation box and PPE	Experienced consultant anesthesiologists with at least 10 intubations performed using an intubation box	C‐MAC vs. McGrath	Time to successful intubation	Number of attempts, C‐L grade, POGO score, optimization maneuvers, difficulty of device use, oropharyngeal morbidity, user preference
3.	10.1093/bja/aew058	Exclusion of patients with known or suspected difficult airway. Difficulty caused by the placement of a cervical collar, which limited mouth opening and neck movement	Consultant anesthesiologists	Three channeled videolaryngoscopes (Airtraq, A.P. Advance, and King Vision) vs. three nonchanneled videolaryngoscopes (C‐MAC D‐blade, GlideScope, and McGrath blade no. 3)	First‐pass intubation success	Overall intubation success, C‐L grade, POGO score, difficulty scale, number of attempts, adverse events, reasons for failure, operator preference
4.	10.1093/bja/aes145	Mallampati score ≥ 3. Exclusion of patients with other difficult‐intubation criteria	Experienced anesthesiologists	McGrath vs. C‐MAC, blades no. 3/4	Time to successful intubation	C‐L grade, number of attempts, ratio of successful to failed intubations, ease of intubation, hemodynamic changes, complications
5.	10.1111/anae.13413	Manikin intubation.	Anesthesiologists, anesthesia residents, paramedical personnel, and medical students	Macintosh laryngoscope vs. Airtraq vs. C‐MAC vs. VLP‐100 vs. C‐MAC D‐blade vs. GlideScope vs. McGrath Series 5 vs. Pentax Airway Scope	Time to successful intubation.	Number of attempts, number of optimization maneuvers, C‐L grade, number of device–tooth contacts, user satisfaction
6.	10.1155/2016/8943931	Manikin intubation with a normal airway scenario and a difficult airway scenario with tongue edema	Medical students.	C‐MAC vs. McGrath vs. Macintosh laryngoscope (blade no. 3 for all devices)	Time to intubation	Number of attempts, C‐L grade, difficulty of device use
7.	10.4103/sja.sja_1058_20	Manikin intubation using an intubation box and PPE	Resident physicians with no prior experience in intubation using a videolaryngoscope.	C‐MAC vs. McGrath blade no. 4	Time to intubation	POGO score, C‐L grade, dental trauma, optimization maneuvers, failed intubations, multiple user attempts
8.	10.1016/j.jclinane.2015.12.042	BMI ≥ 30 kg/m^2^; exclusion of patients with a history of difficult intubation	Anesthesiologists with prior training in each of the videolaryngoscopes used	Macintosh laryngoscope vs. Video‐MAC VL vs. GlideScope vs. McGrath VL	Reduction in intubation time when using a videolaryngoscope compared to the conventional laryngoscope	Differences between devices, adverse events, upper‐airway morbidity, hypoxemia
9.	10.1007/s10877‐021‐00746‐5	Exclusion of patients with a history or prediction of difficult airway	Anesthesiologists with 25 years of experience and familiarity with the use of the McGrath X‐blade and the C‐MAC	McGrath X‐blade vs. C‐MAC	First‐pass intubation success	Overall intubation success, number of attempts, time to intubation, C‐L grade, hemodynamic response, desaturation, adverse events

*Note:* C‐L grade = Cormack–Lehane classification; POGO score = percentage of glottic opening.

Abbreviation: BMI = body mass index.

Table 1 summarizes key demographics of the included studies: DOI, first author, publication year, journal, study type, participant count, main intervention, and funding or conflicts of interest.

Table 2 summarizes the clinical characteristics of the included studies, listing for each: airway difficulty, operator experience, laryngoscope type, and primary and secondary outcomes, ordered by study DOI.

The risk of bias was determined to be low across all evaluated domains, including randomization, allocation concealment, blinding, incomplete outcome data, and selective reporting. The principal limitation identified in these studies was the impossibility of blinding the anesthetists to the specific videolaryngoscope being utilized for each patient, which presents a potential for bias due to any preexisting preferences for certain devices. Nonetheless, we consider it highly improbable that this lack of operator blinding resulted in any meaningful deviation from the intended study procedures.

In addition, many studies do not specify whether participants knew which videolaryngoscope group they were in; however, since general anesthesia was used, it is assumed patients could not have identified the device during intubation.

**FIGURE 2:**
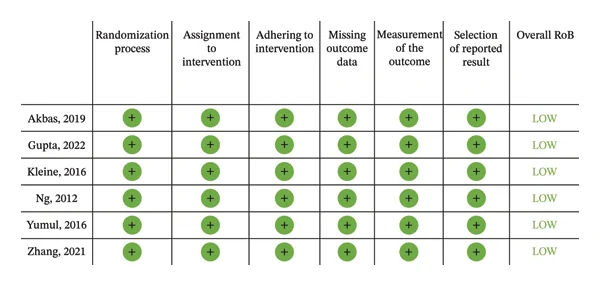
Risk of bias assessment.

Therefore, the overall risk of bias for all studies included in the meta‐analysis is considered low (Figure 2).

### 3.3. Population

The included evidence was strictly separated according to study setting and population. Six studies enrolled adult clinical patients and were eligible for quantitative synthesis, with 780 adult clinical patients who contributed extractable paired data to the quantitative meta‐analysis of C‐MAC versus McGrath outcomes. Within the adult clinical patient studies, two studies enrolled patients with obesity or morbid obesity (BMI > 30 or BMI ≥ 40) [25, 32]; one study evaluated intubation in adult patients under COVID‐19‐related procedural constraints using an intubation box and personal protective equipment [26]; and three studies enrolled adult patients with normal, potentially difficult, or simulated difficult airways created in real patients by study conditions such as cervical immobilization [27, 28, 33]. Three additional studies were manikin‐based airway studies and were retained only for qualitative synthesis [29–31]. These manikin‐based studies included 322 operators or manikin‐based intubation datasets and were not counted as clinical patients. Therefore, the review should be described as enrolling 780 adult patients, with adult clinical patient data and manikin‐based simulation data being reported separately, and only adult clinical patient studies being pooled quantitatively.

### 3.4. Intervention/Comparison

The study examined C‐MAC and McGrath videolaryngoscopes for endotracheal intubation. As such, the intervention was accomplished in five studies [25, 26, 28, 31, 33]. Two studies [27, 29] compared more than two videolaryngoscopes, including the ones under investigation, while two other studies [30, 32] compared them to the conventional Macintosh laryngoscope as well. We compared the devices based on time to intubation and first‐pass success rates.

### 3.5. Outcome: Qualitative Synthesis

The findings of the included 9 studies [25–33] are presented in Table 3.

**TABLE 3:** Primary and secondary endpoints of the included studies.

A/n	DOI	Devices	Number of patients in each device (*n*)	First‐pass intubation success *n* (%)		Total intubation success *n* (%)		Time to intubation (sec) mean ± SD OR median (IQR)		Hypoxemia (*n*)	Dental trauma (*n*)	Hoarse voice (*n*)	Sore throat (*n*)
1.	10.1089/bari.2018.0052	McGrath	40	37 (92.5%)		40 (100%)		55.2 ± 6.32		0	0	3 (7.5%)	0
		C‐MAC	40	37 (92.5%)		40 (100%)		38.65 ± 17.57		1 (2.5%)	0	0	1 (2.5%)

2.	10.5152/TJAR.2021.21251	McGrath	30	80%		30 (100%)		57.93 ± 14.92		0	0	0	0
		C‐MAC	30	90%		30 (100%)		57.17 ± 19.98		0	0	0	0

3.	10.1093/bja/aew058	C‐MAC	120	114 (95%)		117 (98%)		56 (45.85)		0	0	No statistically significant difference (11%–18%)	No statistically significant difference (10%–19%)
		McGrath	120	117 (98%)		118 (98%)		53 (42.77)		0	0		
		GlideScope	120	102 (85%)		110 (92%)		60 (48.98)		0	0		
		Airtraq	120	102 (85%)		112 (93%)		47 (36.60)		0	0		
		A.P. Advance	120	44 (37%)		48 (40%)		93 (54.144)		0	0		
		KingVision	120	104 (87%)		110 (92%)		59 (46.78)		0	0		

4.	10.1093/bja/aes145	McGrath	65	45 (69%)		60 (92%)		67 (50.110) approx.		N/Ref.	N/Ref.	N/Ref.	N/Ref.
		C‐MAC	65	58 (89%)		64 (98%)		50 (35.60) approx.					

5.	10.1111/anae.13413	Macintosh	Use of each device by: 65 anesthesiologists, 67 residents, 56 paramedical personnel, and 65 medical students.	Not reported		An_CMAC: 65 (100%)		An_CMAC: 12 (9.16) approx.		N/Ref.	The students had higher rates of trauma with the Macintosh compared to the C‐MAC and McGrath.	N/Ref.	N/Ref.
		Airtraq				An_McGrath: 59 (91%)		An_McGrath: 10 (7.13) approx.					
		C‐MAC				Res_CMAC: 67 (100%)		Res_CMAC: 9 (5.12) approx.					
		VLP‐100				Res_McGrath: 60 (95%)		Res_McGrath: 10 (6.14) approx.					
		D‐Blade				Par_CMAC: 50 (89%)		Par_CMAC: 5 (5.7) approx.					
		GlideScope				Par_McGrath:44 (78%)		Par_McGrath: 7 (5.7) approx.					
		McGrath				Stud_CMAC: 65 (100%)		Stud_CMAC: 16 (10.18) approx.					
		Pentax				Stud_McGrath: 48 (73%)		Stud_McGrath: 8 (5.15) approx.					

6.	10.1155/2016/8943931	C‐MAC	39	N/A: 39 (100%)	D/A: 36 (92%)	N/A: 39 (100%)	D/A: 37 (95%)	N/A: 23.2 (20.8‐25.7)	D/A: 32.8 (28.1‐37.5)	N/Ref.	N/Ref.	N/Ref.	N/Ref.
		McGrath	39	N/A: 39 (100%)	D/A: 35 (90%)	N/A: 39 (100%)	D/A: 38 (97%)	N/A: 21.8 (19.4‐24.2)	D/A: 31.7 (27.1‐36.3)				
		Macintosh	39	N/A: 31 (80%)	D/A: 25 (64%)	N/A: 38 (97%)	D/A: 27 (69%)	N/A: 26.6 (24.2‐29.1)	D/A: 34.3 (29.3‐39.3)				

7.	10.4103/sja.sja_1058_20	McGrath	15	14 (96.67%)		14 (96.67%)		31.33 ± 14.72		N/Ref.	4 (13.3%)	N/Ref.	N/Ref.
		C‐MAC	15	15 (100%)		15 (100%)		26.27 ± 8.5			1 (3.3%)		

8.	10.1016/j.jclinane.2015.12.042	Macintosh	31	23 (74%)		31 (100%)		70 ± 43		N/Ref.	N/Ref.	4 (13%)	5 (16%)
		VideoMAC	30	28 (93%)		30 (100%)		49 ± 25				5 (16%)	6 (20%)
		GlideScope	30	28 (93%)		30 (100%)		69 ± 34				1 (3%)	7 (23%)
		McGrath	30	21 (70%)		30 (100%)		62 ± 31				3 (10%)	11 (36%)

9.	10.1007/s10877‐021‐00746‐5	McGrath X‐blade	105	75 (71.4%)		96 (91.4%)		55.5 (42.1–78.3)		0	0	N/Ref.	N/Ref.
		C‐MAC	105	83 (79%)		97 (92.5%)		43.8 (38–55.3)		0	0		

*Note:* An_CMAC, An_McGrath = anesthesiologist group; Res_CMAC, Res_McGrath = resident group; Par_CMAC, Par_McGrath = paramedic group; Stud_CMAC, Stud_McGrath = Student group; approx. = approximately; N/Ref. = no reference.

Abbreviations: D/A = difficult airway; N/A = normal airway.

Considering first‐pass success rate, in one study [25], the two devices had the same success rate (92.5%). In another study [29], first‐pass intubation success rate was not reported. Out of the remaining seven studies, one found higher rates with the McGrath [27], whereas the others showed a slight favor over C‐MAC [26, 28, 31–33], with one study [30] showing the same success rates in adults with normal airway between C‐MAC and McGrath (100%), with a slight supremacy of the C‐MAC when difficult airway was presented (92% over 90% success of the McGrath).

Regarding time to intubation, almost all nine studies showed a favor over C‐MAC. In five studies [25, 28, 31–33], the use of C‐MAC showed significantly lower times to successful intubation in comparison to McGrath. In three studies [26, 29, 30], intubation times with the two devices were similar, although in the study by Pieters et al. [29], it appeared there was a significant reduction in time to intubation in the group of students who used the McGrath device, as appeared in the study of Shin et al. [30], where the McGrath showed a slight supremacy in the normal airway group. Lastly, one study [27] showed faster time to intubation with the McGrath (53 s compared to 56 s with the C‐MAC). It is important to recognize that manikin‐based airway studies do not have the same clinical relevance as adult clinical patient studies, because they lack physiological responses and real‐world clinical complexity. Consequently, findings from manikin‐based airway studies should not be weighted equally with adult clinical patient randomized trials in this systematic review. In addition, the incidence of airway management complications, such as hypoxemia, dental trauma, hoarse voice, and sore throat, is presented in Table 3 for each study included in the systematic review, but because of the lack of data in many of the studies, these parameters were not included in the quantitative synthesis.

### 3.6. Quantitative Synthesis

Quantitative synthesis included only adult clinical patient studies with extractable paired C‐MAC versus McGrath data. The six studies included in the meta‐analysis were Akbas et al. [25], Gupta et al. [26], Kleine‐Brueggeney et al. [27], Ng et al. [28], Yumul et al. [32], and Zhang et al. [33]. No manikin‐based airway studies were included in any pooled quantitative analysis. The manikin‐based studies by Pieters et al. [29], Shin et al. [30], and Vig et al. [31] were excluded from quantitative pooling and were summarized qualitatively only. Therefore, the number of 780 patients refers exclusively to adult clinical patients and does not include manikin intubations, operators, or simulation datasets. Two clinical studies identified first‐pass intubation success as their primary endpoint [27, 33], three clinical studies defined intubation time as their primary endpoint [26, 28, 32], and one clinical study reported both first‐pass success and intubation time as primary endpoints [25]. Data were evaluated using fixed‐effect models when heterogeneity estimates were low and random‐effects models when heterogeneity was moderate or high.

### 3.7. First‐Pass Intubation Success Rate

**FIGURE 3:**
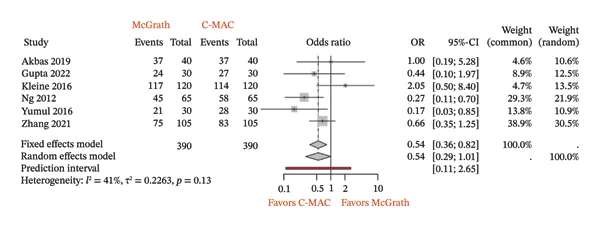
Forest plot for first‐pass intubation success when all studies are included in the meta‐analysis.

A total of 780 adult clinical patients from six adult clinical patient studies—Akbas et al. [25], Gupta et al. [26], Kleine‐Brueggeney et al. [27], Ng et al. [28], Yumul et al. [32], and Zhang et al. [33]—were included in the meta‐analysis of first‐pass intubation success, with 390 allocated to McGrath and 390 allocated to C‐MAC. These were clinical patient data only; no manikin‐based airway studies, simulation datasets, or operator‐only datasets were included in this pooled estimate. As illustrated in Figure 3, analysis with the random‐effects model showed no statistically significant difference in first‐pass intubation success between the two devices (OR: 0.54, 95% CI: 0.29–1.01). The fixed‐effects model indicated a significant advantage for C‐MAC (OR: 0.54, 95% CI: 0.36–0.82), but because heterogeneity was moderate (I^2^ = 41%), the random‐effects model was considered more appropriate. Therefore, the primary interpretation is that no definitive difference was demonstrated for first‐pass success in adult clinical patients.

**FIGURE 4:**
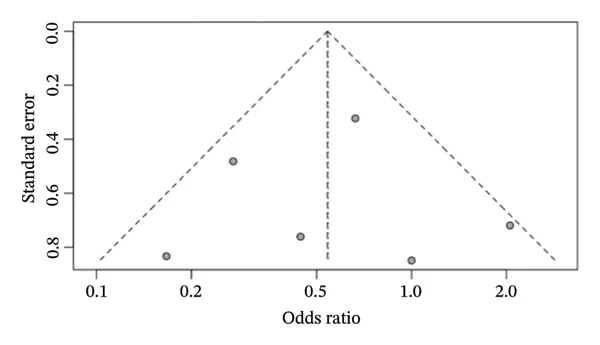
Funnel plot for publication bias regarding first‐pass intubation success when all studies are included in the meta‐analysis.

The prediction interval (0.11–2.65) suggests that the meta‐analysis results remain robust and reliable with respect to future studies. The studies by Zhang [33] and Ng [28] exerted the greatest influence, contributing 30.5% and 21.9% of the weighting in the random‐effects model and 38.9% and 29.3%, respectively, in the fixed‐effects model. The funnel plot was symmetrical, indicating no significant publication bias (Figure 4), and the Egger test showed no statistically significant result (*z* = 0.42, *p* = 0.67). It is important to acknowledge that publication bias tests are generally considered robust when a substantial number of studies—typically more than ten—are included in the meta‐analysis. Given the smaller sample size in this analysis, the results of funnel plots and Egger’s tests concerning publication bias should not be used to draw definitive conclusions.

**FIGURE 5:**
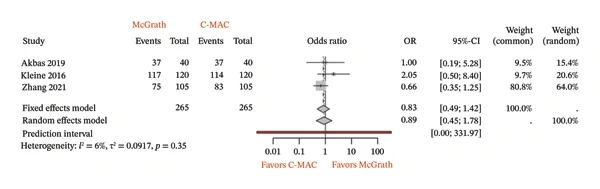
Forest plot for the sensitivity analysis regarding first‐pass intubation success.

**FIGURE 6:**
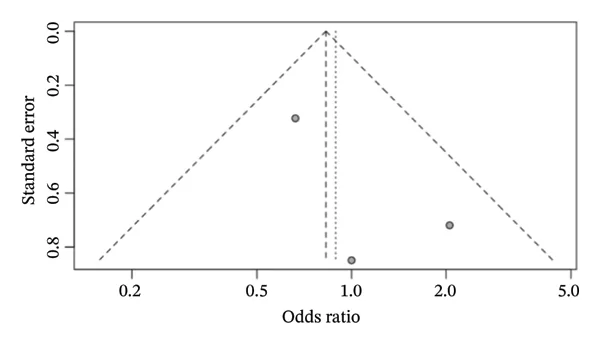
Funnel plot for publication bias for the sensitivity analysis regarding first‐pass intubation success.

To test the robustness of the first‐pass success meta‐analysis, we performed a sensitivity analysis by excluding studies with a different primary endpoint than first‐pass intubation success. After exclusion of the three studies that identified time to intubation as their primary outcome [26, 28, 32], no statistically significant difference was observed in first‐pass intubation success between the two videolaryngoscopes in the sensitivity analysis. The OR under the fixed‐effects model was 0.83 (95% CI: 0.49–1.42), and the random‐effects model yielded comparable results (OR: 0.89; 95% CI: 0.45–1.78). Heterogeneity was minimal, as indicated by I^2^ = 6%, tau^2^ = 0.0917, *H* = 1.03, and *Q* = 2.12 (*p* = 0.35), suggesting a very low degree of between‐study variability in the sensitivity analysis (Figure 5). The prediction interval (0.00–331.97) further suggested that the results of the meta‐analysis remain robust with respect to future research. The sensitivity analysis confirms the random‐effects model, showing no significant difference in first‐pass intubation success between the two videolaryngoscopes when all studies are combined. The funnel plot demonstrated symmetry, indicating no apparent publication bias (Figure 6), although it cannot be reliable for concrete conclusions.

### 3.8. Time to Intubation

**FIGURE 7:**
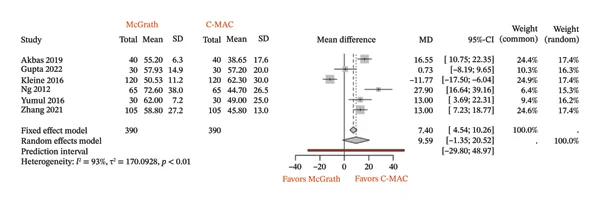
Forest plot regarding time to intubation when all studies are included in the meta‐analysis.

A total of 780 adult clinical patients from the same six adult clinical patient studies—Akbas et al. [25], Gupta et al. [26], Kleine‐Brueggeney et al. [27], Ng et al. [28], Yumul et al. [32], and Zhang et al. [33]—were included in the meta‐analysis of time to intubation, comprising 390 individuals intubated with McGrath and 390 with C‐MAC. These data were derived exclusively from adult clinical patient studies; manikin‐based studies were not included. The forest plot in Figure 7 showed no statistically significant difference between the devices using the random‐effects model (MD: 9.59 s, 95% CI: −1.35–20.52). The fixed‐effects model suggested a shorter intubation time with C‐MAC (MD: 7.40 s, 95% CI: 4.54–10.26), but heterogeneity was substantial (I^2^ = 93%). Accordingly, the random‐effects model was prioritized, and the pooled result should be interpreted as uncertain rather than definitive for adult clinical patients.

**FIGURE 8:**
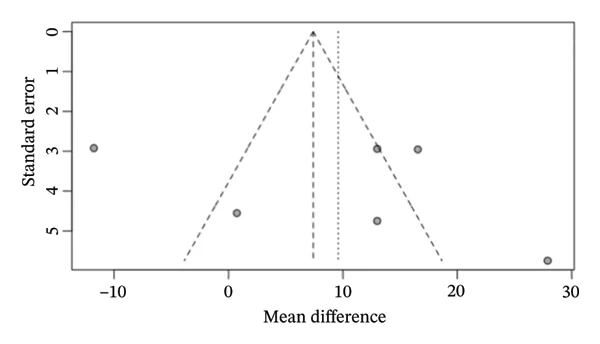
Funnel plot for publication bias regarding time to intubation when all studies are included.

The prediction interval (−29.80–48.97) suggested that the results of the meta‐analysis remain robust with respect to the influence of future studies. The studies by Zhang [33], Akbas [25], and Kleine [27] had the greatest influence, each contributing 17.4% to the random‐effects model and 24.6%, 24.4%, and 24.9%, respectively, to the fixed‐effects model. The asymmetry observed in the funnel plot indicates the presence of substantial publication bias (Figure 8), which is further supported by the Egger’s test (*t* = 5.4603, *p* = 0.0055). It is important to reiterate that, because only a small number of studies were included, the funnel plots for publication bias (both for the overall study and the sensitivity analysis mentioned as follows), along with Egger’s tests, cannot be relied on for definitive conclusions since their interpretation is limited.

**FIGURE 9:**
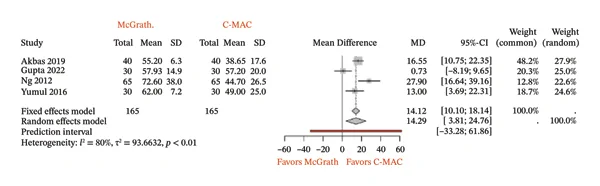
Forest plot for the sensitivity analysis regarding time to intubation.

To assess its impact on the meta‐analysis, a sensitivity analysis excluded studies with first‐pass intubation success as their primary outcome. After exclusion of the two major studies with that endpoint—Kleine [27] and Zhang [33]—both the fixed‐effects and random‐effects models demonstrated a significant advantage in favor of the C‐MAC (MD: 14.12, 95% CI: 10.10–18.14 for the fixed‐effects model and MD: 14.29, 95% CI: 3.81–24.76 for the random‐effects model). The substantial heterogeneity observed among the included studies was reflected by an I^2^ of 80%, tau^2^ of 93.66, H of 2.25, and a Q value of 15.13 (*p* < 0.0017). Therefore, the random‐effects model was considered more appropriate for the analysis of these data, indicating a significant difference between the two devices. Specifically, intubation with the C‐MAC was, on average, 14.29 s faster (Figure 9).

**FIGURE 10:**
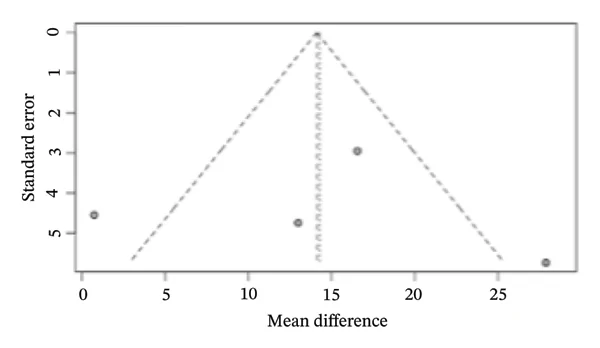
Funnel plot for publication bias for the sensitivity analysis regarding time to intubation.

The prediction interval (−33.28–61.86) indicates that the meta‐analysis results are likely to hold despite potential future studies. The studies by Akbas [25], Gupta [26], and Yumul [32] were the most influential, contributing 27.9%, 25.0%, and 24.6%, respectively, to the random‐effects model. Funnel plot asymmetry indicates the presence of significant publication bias (Figure 10), always given its limited interpretability.

Both sensitivity analysis and random‐effects model results showed that the C‐MAC was faster for intubation. However, high heterogeneity values (I2 = 93% and I2 = 80%) indicate a significant limitation affecting the reliability of these pooled estimates.

### 3.9. Summary of Findings and Certainty of Evidence Assessment

In accordance with PRISMA recommendations, certainty of evidence for the main outcomes was assessed using a GRADE‐based approach and summarized in a dedicated outcomes table (Table 4). Only adult clinical patient studies were included in the certainty assessment for pooled quantitative outcomes. Manikin‐based airway studies and simulation datasets were not incorporated into the pooled certainty judgments because they represent indirect evidence for clinical patient care. Evidence from randomized clinical studies was initially considered high certainty and was downgraded for inconsistency, imprecision, indirectness, and concern for small‐study or publication effects when applicable.

**TABLE 4:** Summary of findings and certainty of evidence assessment for adult clinical patient outcomes.

Outcome	Population and setting	Studies included in pooled analysis	Participants included	Effect estimates	Certainty of evidence	Reasons for downgrading	Interpretation
First‐pass intubation success	Adult clinical patients only; manikin‐based and simulation studies excluded from pooling	Akbas et al. [25], Gupta et al. [26], Kleine‐Brueggeney et al. [27], Ng et al. [28], Yumul et al. [32], and Zhang et al. [33]	780 adult clinical patients	OR: 0.54; 95% CI: 0.29–1.01 using the random‐effects model	Low	Downgraded for moderate inconsistency and imprecision because the confidence interval approached no effect and between‐study variability was present	No definitive difference between C‐MAC and McGrath was demonstrated for first‐pass success in adult clinical patients
Time to intubation	Adult clinical patients only; manikin‐based and simulation studies excluded from pooling	Akbas et al. [25], Gupta et al. [26], Kleine‐Brueggeney et al. [27], Ng et al. [28], Yumul et al. [32], and Zhang et al. [33]	780 adult clinical patients	MD: 9.59 s; 95% CI: –1.35 to 20.52 using the random‐effects model	Very low	Downgraded for substantial heterogeneity, imprecision, and concern for small‐study or publication effects	The pooled estimate was uncertain; although some analyses favored C‐MAC, heterogeneity limits confidence in a clinically reliable difference

## 4. Discussion

### 4.1. Summary of Evidence

This meta‐analysis evaluates the clinical effectiveness of C‐MAC and McGrath videolaryngoscopes for adult endotracheal intubation. Both devices are commonly used in anesthesiology and critical care, but their comparative strengths and weaknesses are still under discussion.

Both devices examined in this review offer significant advantages over many other videolaryngoscopes available, including previously reported first‐pass intubation success rates of 98% for the McGrath and 95% for the C‐MAC D‐blade. They also provide excellent overall success rates, superior laryngeal visualization, and quick intubation times [27].

Previous published studies have compared either the C‐MAC or the McGrath videolaryngoscopes with the Macintosh laryngoscope in different patient groups. In the study of Abhyankar et al. [34], the two videolaryngoscopy devices provided a superior glottic view and better first‐pass success rates when compared to the Macintosh laryngoscope. The C‐MAC demonstrated superior intubation characteristics when the two were compared. In the study of Karadağ et al. [35], the C‐MAC and the McGrath videolaryngoscopes were compared with stylet assistance in obese patients. Both methods were effective, but using a stylet improved the intubation success rate.

The results of the present meta‐analysis reveal a complex picture, with findings differing according to the chosen endpoint and analytical approach. Regarding first‐pass intubation success, the random‐effects model applied to all included studies did not identify a statistically significant difference between the two devices. A prior meta‐analysis found that reliable comparisons between videolaryngoscopes in adult general anesthesia are not possible due to low evidence certainty [36]. This analysis, in line with previous reviews, does not show strong evidence that either device is better for achieving first‐pass success. However, there was moderate variability among the studies (I^2^ = 41%), which could be due to differences in study participants, operator skill, how the devices were used, and procedural methods, as mentioned in the following. Sensitivity analysis excluding studies with intubation time as the primary endpoint showed no significant difference in first‐pass intubation success between the two devices, consistent with the overall meta‐analysis.

Regarding intubation time, the overall meta‐analysis indicated that the C‐MAC was linked to a marginally shorter intubation duration in the sensitivity analysis; however, this difference did not reach statistical significance, as evidenced by the 95% confidence interval of −1.35–20.52 in the random‐effects model. A previous meta‐analysis by Lee et al. [36] also showed that C‐MAC performed better than other videolaryngoscopes across airway conditions, supporting current results. Sensitivity analysis found that C‐MAC intubated about 14 s faster than McGrath, though high heterogeneity (I^2^ = 80%) limits firm conclusions. Clinically, this time difference is negligible for routine intubations but may be more significant in challenging cases. Still, caution is needed in interpreting these findings.

### 4.2. Interpretation of Findings

Careful consideration is necessary when interpreting these inconsistent findings. Several variables may contribute to the differences observed among the studies, listed as follows.

#### 4.2.1. Population Heterogeneity

Variations in patient characteristics—such as underlying disease severity, BMI, and airway anatomy (e.g., Mallampati score)—can impact intubation difficulty and may make one device preferable over another. The C‐MAC D‐blade, for instance, tends to be particularly effective for patients with restricted neck mobility or elevated Mallampati scores. This efficacy is attributed to the D‐blade’s pronounced angulation (40° compared to 18° for the standard C‐MAC blade), which improves glottic visualization, along with a purpose‐designed hyperangulated stylet that aids endotracheal tube placement in complex airways. These mechanical distinctions between devices may help account for the superior intubation times reported with the C‐MAC D‐blade.

#### 4.2.2. Operator Experience

Clinician familiarity with each device can affect intubation time and first‐pass success. Some anesthesiologists work faster with the C‐MAC because it resembles direct laryngoscopy, while others prefer the McGrath for its lightweight and ergonomic design. Differences in training and routine practice may explain study heterogeneity.

#### 4.2.3. Technical Factors

Variations in intubation techniques—such as using a stylet, applying external laryngeal manipulation, or choosing direct or indirect visualization of the airway—can have a major impact on how procedures turn out. Because these specific methods are not always the same from study to study, they may explain differences in reported results.

#### 4.2.4. Clinical Setting

The relative effectiveness of the two videolaryngoscopes can change depending on conditions; results in a controlled operating room may not apply to emergency or out‐of‐OR situations due to factors such as time constraints, patient positioning, and environmental challenges.

### 4.3. Limitations of the Study

Although this meta‐analysis was conducted systematically and thoroughly, several limitations must be acknowledged to ensure accurate interpretation of the findings and inform future research design. First, the number of studies meeting the inclusion criteria was limited, with only six out of nine eligible studies included. This restricts the statistical power of the analysis and may reduce sensitivity to detect subtle but meaningful differences between the two videolaryngoscopes. The small sample of studies also impacts predictive accuracy and limits generalizability to broader patient populations. Consequently, the results should be interpreted cautiously due to these constraints.

A key limitation is heterogeneity among the clinical studies, as indicated by I^2^ values of 41% for first‐pass intubation success and 93% for intubation time. Sources of heterogeneity include differences in BMI, airway difficulty, cervical immobilization, COVID‐19‐related barriers, operator experience, blade configuration, use of stylets or optimization maneuvers, and definitions of outcomes. To reduce inappropriate clinical interpretation, simulated‐patient and manikin‐based airway studies were not included in the quantitative meta‐analysis. These studies remain useful for evaluating training, ergonomics, and device handling under standardized conditions, but they do not reproduce physiological responses, secretions, airway trauma, desaturation risk, or the full complexity of clinical intubation.

Another concern is the possibility of publication bias. Reliable publication bias assessment requires a minimum of 10 studies; with only six or fewer included here, such tests are not dependable for drawing firm conclusions. It should be noted that these analyses are exploratory. The literature search was restricted to English‐language publications, potentially excluding relevant studies published in other languages. Data were unavailable for some secondary endpoints, such as trauma and bleeding, precluding meta‐analysis on these outcomes. In addition, reliance solely on published study data, without access to primary patient records, limits deeper subgroup analyses and more sophisticated modeling. Finally, individual study limitations—such as lack of blinding—may persist within the meta‐analysis, although assessment with the Cochrane Risk of Bias 2.0 tool suggests minimal risk of additional bias from this factor. However, when the attending anesthesiologist is aware of the device in use, this awareness may introduce performance bias, as well as differences in skill application and familiarity with each device, thereby potentially affecting outcomes such as first‐pass intubation success and intubation time.

One final limitation of this study is the overlap in definitions for “first‐pass intubation success rate” and “time to successful intubation” among the included studies. Some studies defined time to intubation as the interval from the introduction of the laryngoscopy blade into the oral cavity until the initial detection of end‐tidal CO_2_ waveform, while others considered it to be the period from blade insertion between the teeth to effective ventilation with an Ambu bag. Similarly, definitions of an attempt varied; certain studies described it as a single advancement of the styleted tracheal tube toward the glottis, whereas others considered it as insertion of the laryngoscopy blade into the patient’s mouth, irrespective of subsequent tube placement attempts. Not every study provided a definition of success; however, one investigation specified success as placement of the tube in the trachea within 180 s, confirmed by the appearance of end‐tidal CO_2_ on the waveform.

These limitations should be considered when evaluating the findings. The results regarding first‐pass intubation success and intubation time show some inconsistency. While sensitivity analysis suggested a trend favoring the C‐MAC device for intubation time, overall results revealed no statistically significant differences between the C‐MAC and McGrath MAC video laryngoscopes in either outcome. Nevertheless, this meta‐analysis contributes an overview of their comparative effectiveness and identifies areas for future investigation. Addressing these limitations in subsequent research will enhance understanding of the relative merits of both devices and support the optimization of airway management practices.

### 4.4. Future Research

The above limitations highlight the need for robust future research to better assess these videolaryngoscopes. Well‐designed, prospective RCTs comparing the devices are necessary, with strict selection criteria for homogeneous patient groups and detailed control of variables such as airway history, BMI, Mallampati classification, comorbidities, and prior intubations. Standardized intubation protocols and careful consideration of operator experience, including skill assessments and documentation of training and number of procedures, will help minimize variability and systematic errors.

To improve assessment accuracy, future research should use objective outcomes such as intubation time, Cormack–Lehane scores for vocal cord visualization, and patient oxygenation levels (SpO2). These measures minimize subjective bias and allow clearer device comparisons. Studies should also evaluate costs related to purchasing, maintaining, and training for each video laryngoscope, as well as the financial impact of complications. Comprehensive cost‐effectiveness analysis will aid policymakers and clinical institutions in resource allocation decisions.

Simulated‐patient and manikin‐based airway studies offer a robust approach to evaluating the performance of videolaryngoscopes within controlled and standardized settings. These studies enable assessment of intubation success rates, time to intubation, and the incidence of complications across diverse scenarios, including difficult airway management, emergency interventions, and challenges such as the presence of blood or vomit. Furthermore, simulation‐based research plays a crucial role in the education and training of healthcare professionals, fostering proficiency with these devices prior to their application in clinical practice.

It is also essential to expand investigations into the effectiveness of videolaryngoscopy among specific patient populations—such as individuals with obesity, those with restricted neck mobility, multitrauma patients in emergency departments, and critically ill patients in intensive care units. These cohorts often demonstrate unique anatomical and physiological characteristics that may impact device performance. Future research should focus on enrolling adequate numbers of participants from these groups to ensure sufficient statistical power and generate reliable findings.

In addition, the adoption of multivariate research designs is recommended to elucidate the complex interplay of factors influencing intubation outcomes. Such approaches allow simultaneous evaluation of variables such as operator experience, patient‐specific attributes, and procedural techniques. By analyzing these interactions, researchers can achieve a more comprehensive understanding of the circumstances under which one video laryngoscope may outperform another.

## 5. Conclusions

In this systematic review and meta‐analysis, adult clinical patient evidence did not demonstrate definitive superiority of either the C‐MAC or McGrath videolaryngoscope for first‐pass intubation success. C‐MAC was associated with shorter intubation time in some analyses, but this finding was limited by substantial heterogeneity and very low certainty of evidence. Simulated‐patient and manikin‐based airway studies were useful for contextualizing device handling and training but were not pooled with adult clinical patient data. Overall, the findings should be interpreted cautiously and should not be generalized across adult clinical patient, simulated‐patient, and manikin‐based airway settings. Future randomized trials should use homogeneous patient populations, standardized intubation protocols, clearly defined outcomes, and prespecified subgroup analyses to determine whether either device offers clinically meaningful advantages in specific airway contexts.

**Nomenclature**
DAS: Difficult Airway Society
ASA: American Society of Anesthesiologists
PICO: Population, intervention, comparison, and outcomes
DOI: Digital object identifier
OR: Odds ratio
CI: Confidence interval
MD: Mean difference
I^2^
: Heterogeneity index
Tau^2^
: Between‐study variance index
H: Heterogeneity
SE: Standard error
VL: Videolaryngoscope
C‐L grade: Cormack–Lehane grade
POGO score: Percentage of glottic opening score
BMI: Body mass index
N/A: Normal airway
D/A: Difficult airway
An_CMAC, An_McGrath: Anesthesiologist group
Res_CMAC, Res_McGrath: Resident group
Par_CMAC, Par_McGrath: Paramedic group
Stud_CMAC, Stud_McGrath: Student group
approx.: Approximately
N/Ref: No reference
RCT: Randomized controlled trial
OR: Operating room

## Author Contributions

All authors have made substantial contributions to all parts of this study. Evgenia Dimou and Theodoros Xanthos designed the study and drafted the manuscript. Evgenia Dimou and Diamanto Aretha extracted and analyzed the data. Evgenia Dimou, Theodoros Xanthos, Theano Beskou‐Kontou, Antonis Kostouros, Matthaios Pappas, and Angeliki Panagopoulou searched and selected the articles. Evgenia Dimou, Theodoros Xanthos, and Diamanto Aretha critically revised the manuscript.

## Funding

No funding was received for this study.

## Disclosure

All authors have approved the final version and agree to be accountable for all aspects of the work, ensuring integrity and accuracy.

## Conflicts of Interest

The authors declare no conflicts of interest.

## Supporting Information

Additional supporting information can be found online in the Supporting Information section.

## Supporting information


**Supporting Information** The PRISMA 2020 checklist is a reporting guideline designed to help researchers transparently and completely report systematic reviews and meta‐analyses. It comprises an expanded checklist that details reporting recommendations for each item and subitem included in the review, improving the clarity and reproducibility of systematic review reports [23] (Supporting Information).

## Data Availability

All data used to support the findings of this study are available from the corresponding author on reasonable request.
